# Editorial

**Published:** 1871-06

**Authors:** 


### Editorial.

### CORRESPONDENCE OF THE SENIOR EDITOR.

On Monday morning, April 24th, 1871, at 10.45, we took seats in one of Mr. Pullman's elegant palace sleeping cars, surrounded by as pleasant a company of gentlemen and ladies as ever traveled together, and started across the Continent for the City of the Golden Gate. During all that day and night we were passing over the rich and familiar prairie country of Northern Illinois and Iowa, through which the Fulton and Omaha branch of the North-Western Railroad passes, and reached Omaha about 9 o'clock A.M. of the second day. There we renewed our accommodations, and were soon again moving majestically onward towards the great mountain chain that divides the waters of the Mississippi from those that flow into the Pacific Ocean. During the remainder of that day and night, and all the following day, our course was onward and upward; but the ascent was so gradual, and the mountain slopes spread out on so grand a scale, that we could hardly realize either the distance or the degree of elevation. During the forepart of the third day we were passing over those almost boundless plains, inhabited by buffaloes, antelopes, wolves, and prairie dogs. Of the antelope and prairie dogs we saw plenty, and imagined that we saw a small herd of buffaloes at a distance. In the afternoon, while rapidly approaching the highest points on the road, we first found the ground partially covered with snow, and were soon after enveloped in a driving snowstorm—not lasting long, however, and making no barrier to our progress. After passing the highest point of the whole route, being little more than 8000 feet (at Shermon), and reaching the plain dividing the waters flowing eastward into the

Platte from those flowing southward through the Green River into the Colorado, night again obscured our vision, caused our seats to be converted into beds, and we gladly exchanged the wonders of Nature and the brilliant conversazione of our party for sweet sleep and the fairy-land of dreams. At early dawn, taking our station on the platform—while most of our companions were still in their berths—we found the train just crossing the dividing ridge between the Green River basin and the head waters of the Bear River, which empties into Salt Lake. This Green River and Bitter Creek plain or basin is the first (as we go westward) of a series of vast and elevated plains, having all the appearance of having been once great inland seas. It is bounded on the east by the Rocky Mountain chain proper, and on the west by the Wasachts, while both north and south the eye constantly rests on mountain peaks, many of which are covered with snow. It is mostly barren, and its waters so bitter and alkaline, that they are not only unfit for man or beast, but they cannot be used to supply the engines running on the road. Yet its geological features are interesting, and it contains a valuable stratum of coal, which is already being worked, to supply the fuel for use along the railroad. Crossing the Bear River, we were quickly engaged in the rugged and snow-capped Wasacht range of mountains, and after many curves, striking the head waters of Webber River, we followed its clear but turbulent waters through one of the grandest mountain gorges to be found on the entire route. It is here that we pass through the celebrated Echo Canyon and Devil's Gap, and enjoy every variety of mountain scenery, from the terrific gorge with its overhanging cliffs; the grand mountain with its towering and barren rocks; to the green and gentle slope decked with flowers. As we emerge from the gap in the Wasacht, near the mouth of Webber River, Salt Lake, with its promontory island and surrounding valley, comes suddenly before us, and we are at Ogden, where a good supper and a change from the Union to the Central Pacific Railroad awaits us. These things attended to, and a temporary good-bye to a part of our company who stopped over a day to visit the Salt Lake City, and we were again in motion—not on the sudden curves and rapid grades of the mountain, but across the northern border of Salt Lake Valley, with the placid lake on the one side, and the rugged, snow-capped Wasacht on the other.

We soon reached the new and rapidly-growing town of Corrinne, near the mouth of Bear River, where we saw a small steamboat in process of building to run on Salt Lake. Soon after passing this place night spread his curtain over us, and our company of living freight had their seats converted into beds, and we slept soundly while rolling over the rest of this second great basin or valley. At the first dawn of the morning we had passed the ridges which constitute its western boundary, and were passing smoothly over the first part of the Humboldt Valley, better known as the Great Desert. This is an immense elevated plain, with the naked Humboldt Mountains for its circumference; a river of the same name in its centre, clothed with no vegetation but a little stunted sage brush and a few bunches of bunch grass just on the margin of the river, and a few squalid Indians for its inhabitants. And yet here we go at ordinary railroad speed for thirty-six hours, over a surface more level than the prairies of Illinois. At only one point is the monotony relieved by the mountains on either side approximating until there is only space for the river and the railroad track to pass between the lofty and perpendicular columns of rock, properly called the palisades. Yet over all this desert, and, indeed, over all the route, the stations are well supplied, and the regular eating places furnish as good meals as are to be had anywhere between Chicago and New York. The principal streams flowing into this valley are the Humboldt, the Carson, and the Truckee rivers, all of which end in lakes, that, like the Salt Lake, have no visible outlet, the water doubtless disappearing mostly by evaporation. The whole region is strongly alkaline, and the waters of the Humboldt and Carson rivers are so bitter as to render the fish and fowls that inhabit them unfit for food. The Truckee has its origin in the Eastern slope of the Sierra Nevada range of mountains, and coming into the western border of the great desert, empties into Pyramid Lake. As the morning dawned on us, after we had entered the eastern part of the Humboldt valley, so night overtook us before we reached its western terminus, and the following morning found us ascending the first or eastern range of the Sierra Nevada, through the narrow and tortuous gorge made by the Truckee. In every direction the mountains rise abrupt, rugged, rocky, and sparsely covered with the red wood and pine, while the turbulent water of the Truckee was foaming and dashing along the rocky passage below. A rapid up grade, so tortuous as to turn the engine towards all points of the compass within an hour, soon brought us into the long snow-shed (more than 30 miles in length) that shelters the track at the summit of the Sierres. There everything was covered with snow from one to two feet deep. After a brief halt we emerged from the snow-shed, and for a time were winding among the mountain summits, looking out upon some of the grandest scenery that the imagination can conceive. So rapid was our descending grade that at breakfast, between eight and nine o'clock in the morning, we had not only left the region of snow, but were surrounded with green fields, opening flowers, and singing birds. After breakfast we resumed our course down the mountains on a grade that falls 3000 feet in a distance of less than 100 miles. About noon we arrived at Sacramento, and after following the river of that name a while we left it, and winding our way through the coast range of hills, arrived at Oakland, on the eastern shore of San Francisco Bay, with the city full in view. A good ferry-boat soon transferred us across the bay, and we found ourselves comfortably quartered at the Grand Hotel, having travelled without breaking connections or halting, except for supplies, from 10.45 on Monday morning until six o'clock on Saturday evening in completing the trip from Chicago to San Francisco.

The topography, geology, and climatology of the route afford topics of great interest, not to the scientific only, but to the physician and sanitarian. A discussion of these, including the topography and climate of San Francisco and its vicinity, must be deferred until another number of the EXAMINER. As I send you full accounts of the proceedings, both of the Association of Medical Editors and of the American Association, it will be unnecssary to make more than a brief allusion to them in this letter. The anniversary meeting of the former was held on Monday, and although but few of those connected with the medical press were present, yet the meeting was an interesting and profitable one. The officers elected for the ensuing year were: B. F. Dawson, M.D., of New York, President; Henry Gibbons, jun., M.D., of San Francisco, Vice-President; and F. H. Davis, M.D., of Chicago, Secretary. The address of the retiring president, Dr. H. R. Storer, was delivered in the evening, and listened to by a good audience. The American Medical Association held its meetings in Pacific Hall. It commenced its session at 11 o'clock A.M. Tuesday, and did not finish its work until Friday evening, The President, Dr. Alfred Stillé, of Philadelphia, presided with ability, and all business was transacted in good order, and with good feeling. We think the number present was about three hundred, embracing representatives from as large a number of States as in most of the previous meetings. The number of reports from special committees, and scientific papers was less than usual; still the sections on climatology and epidemics, surgery and anatomy, practical medicine and obstetrics, and materia medica and chemistry, all had work to do, and the resulting volume of transactions will be sufficiently voluminous. Among the miscellaneous topics of most prominence that engaged the attention of the general meetings of the Association, were the mode of admitting members into the profession, the regulation of medical college fees, and the recognition of female practitioners and institutions for their education. On the first of these topics a report was made by the committee previously appointed to correspond with State medical societies, which was adopted by the Association, and the committee continued; also a paper from Dr. Moses, of St. Louis, which was referred to the committee on medical education. The policy of attempting to regulate the fees of medical colleges, and of admitting to representation in the Association the institutions exclusively for the education of females, was discussed with more fulness, freedom, and courtesy than at any previous meeting. These topics have been persistently urged upon the attention of the Association by a few of its members, for the last three or four years. After a fair consideration, the Association decided by a very large majority to leave the subject of college fees with the colleges themselves, wisely considering it far more important to determine how much medical knowledge the student acquires than how much he pays for it.

The question of recognizing females and female institutions turned mostly on the broad ground of whether it is proper or best to directly encourage females to enter upon the study and practice of medicine. After a full discussion the whole subject was negatived by an indefinite postponement. The question was taken by yeas and nays, delegates only voting, and resulted in 80 to 25.

The social entertainments during the week were admirably arranged. Small companies were taken out to the Cliff for breakfast every morning, a most interesting place on the Pacific, affording a view of the ocean, the rocks, seals, etc., with a beautiful drive. On Friday afternoon there was a most pleasant excursion and entertainment by the hospitality of the profession and citizens of Oakland, a rich and beautiful town on the east side of the bay. The whole of Saturday was occupied with a most delightful excursion in a steamer around the bay, passing the Golden Gate, stopping at Mara Island long enough to visit the navy yard, the Marine Hospital, the beautiful flowers, &c., and returning to the city at six in the evening. It was most admirably conducted on behalf of the profession of San Francisco, and was greatly enjoyed by a large company of gentlemen and ladies. But I must close this rambling letter, with the conviction that the meeting in San Francisco has been as successful and pleasant in every respect as the most sanguine could have anticipated.

---

Illinois State Medical Society.—The recent meeting of our State Medical Society, at Peoria, was the largest we have ever attended. The profession and citizens of Peoria received and entertained the Society in the most hospitable and liberal manner. A full account of the proceedings will be given in our next number.

---

IOWA STATE MEDICAL SOCIETY.—The Nineteenth Annual Meeting of the Iowa State Medical Society was held in Des Moines, Iowa, April 19th and 20th, 1871. The number in attendance was unusually large, and all the proceedings were characterized by harmony and the best of feelings. Several valuable papers were read, and verbal reports of special cases, with discussions thereon, contributed to the general interest and improvement. Dr. S. B. Thrall, of Ottumwa, occupied the chair as president *pro tem.*, with Dr. Geo. P. Hanawalt, of Des Moines, as secretary.

The following persons were selected as officers for the ensuing year: President, Dr. A. G. FIELD, Des Moines; Vice-President, Dr. J. M. ROBERTSON, Muscatine; Secretary, Dr. GEO. P. HANAWALT; Corresponding Secretary, Dr. J. F. ELY, Cedar Rapids.

---

MONEY RECEIPTS FROM APRIL 29TH TO MAY 22ND, 1871.—Drs. Peck and Moore, 3.00; Dr. J. B. Newman, 3.00; Dr. W. W. Walton, 6.00; Dr. L. T. Hewins, 3.00; Dr. J. W. Filkins, 3.00; Dr. R. D. Bradley, 3.00; Dr. J. P. Mathews, 3.00; Dr. W. S. Fox, 3.00; Dr. Spickler, 7.25; Dr. Reynolds, 2.75; Dr. C. J. Miller, 3.00; Dr. D. Scott, 5.00; Dr. J. Quirck, 3.00; Dr. J. M. Smith, 2.00; Dr. Daniel Lichty, 3.50; Dr. Wardner, 12.00; Dr. P. P. Gordon, 3.00; Dr. J. W. Barlow, 3.00; Dr. S. P. Breed, 3.00; Dr. W. H. Baxter, 6.00; Dr. T. D. Washburn, 3.00; Dr. Geo. W. Wright, 3.00; Dr. R. E. McVey, 3.00; Dr. J. F. Hamilton, 5.00; Dr. D. Thompson, 3.00; Dr. L. Ware, 3.00; Dr. Wm. Monroe, 10.00; Dr. Dexter, 3.00; Dr. N. W. Abbott, 6.00; Dr. J. M. Baird, 3.00; Dr. L. Tibbets, 3.00; Dr. J. Brewster, 3.00; Dr. G. A. Baunel, 9.00.

---

CARBOLIC ACID IN PRURITUS CUTANEUS.—Professor Binz recently called attention to the value of the internal use of carbolic acid in prurigo and pruritus. He gives it in the form of pills, made up with extract of licorice, containing at first one and a-half grains of the acid, but increasing the dose to fifteen grains per diem. In the latter quantities it sometimes produces gastric disturbances, but which quickly subside when the medicine is given up.—*American Practitioner.*

Hiccough Cured by Chloral.—Dr. P. F. Whitehead has recently prescribed for a patient with hiccough, which had continued for thirty-six hours. Various remedies were used with but little good effect, save a temporary cessation by the use of morphine hypodermically. Thirty grains of chloral gave immediate and permanent relief.—*Medical Record.*

Decline of Vaccination in Great Britain.—Nearly all the British medical journals contain some allusion to the increase of small-pox, which is generally attributed to the decline of vaccination, in consequence of the senseless and fatal prejudice against protection brought about by the anti-vaccination agitators.—*Medical Record.*

Tetanus and Chloral Hydrate.—Dr. George Thompson reports, in the *Richmond and Louisville Medical Journal*, a case of supposed idiopathic tetanus cured by chloral. The dose was 30 grains, repeated several times a day. The cure was effected in four or five days.

Duration of Life.—Mr. Nelson, an English actuary, shows that a temperate person's chance of living is, at 20—44·2 years; at 30—36·5 years; at 40—28·8 years. An intemperate person's chance of living is, at 20—15·6 years; at 30—13·8 years; and at 40—11·6 years.

Deaths from Alcohol in Cities.—Dr. Elisha Harris, of N.Y., states it as his belief, that the annual number of deaths from alcohol in cities is in the proportion of 100 to every 100,000 inhabitants. Of the deaths from disease of the brain, liver, and kidneys, in New York City, in 1869 (1369), twenty per cent., or 273 of these cases, were believed to be the immediate results of alcoholism.—*Proceedings of American Association for Cure of Inebriates.*

Insanity.—Dr. Willard Parker, of N.Y., states that eighty per cent. of the cases of acute insanity treated are restored to health and usefulness.

Intra-Uterine Injections are the subject of much discussion and diversity of sentiment in the profession at the present time. In New York, it is stated that a majority condemn them, thinking they have frequently caused death.—*Pacific Medical Journal.*

Wet Bandages in Fractures.—Guersant (*Medical News and Library*) for all forms of infantile fracture, habitually wets all bandages at the moment of their application, with a resolvent liquid, such as lead-water or camphorated brandy diluted with water, provided the fracture is not complicated with wounds.—*Medical Record.*

The Anterior Splint.—The London Correspondent of the *Baltimore Medical Journal* writes that Smith's anterior splint has been quite extensively introduced in practice in the military hospitals in France since the commencement of the late war by Drs. Tilghman and May, of Baltimore, who were connected with the Anglo-American Ambulance Corps, and have been in constant service since the battle of Sedan. It has been most favorably received, and these surgeons have furnished a number to the Prussian surgeons.

The Carbolic Acid Treatment in the London Hospitals. In these hospitals the carbolic acid treatment, more or less modified, is employed after surgical operations. Mr. Erichsen, of the University College Hospital, washes out the stumps with carbolized water, and applies a very simple dressing of lint wet with it. In a Chopart's operation recently at St. Bartholomew's Hospital, Mr. Paget closed the stump after his operation, accurately, with dressings soaked in a solution of carbolic acid in oil, and said, if all did well, he should allow it to remain so for a week.—*Correspondent Balt. Med. Journal.*

Iodoform Ointment.—In the Boston City Hospital, iodoform ointment in connection with iodide of potassium, is extensively and successfully used in the treatment of syphilitic ulcers and rupia. Dr. William Ingalls, attending surgeon, advocates this formula in two obstinate cases under his care:

℞. Iodoformi ---------------------------ʒss.

Spts. vini. rect. ----------------------q.s.

Adipis suillæ -------------------------ʒvijss. M.

—*Boston Med. and Surg. Journal.*

American Doctors.—According to the present census there are seventy-four thousand doctors in this country.

Cases of Poisoning from Chloral are reported in a great number of the medical journals, both in Europe and America. In most of the cases it has been used without medical advice.—*Pacific Medical Journal.*

### Mortality for the Month of April, 1871.

| | | | | | |
|---|---|---|---|---|---|
| Accidents, burns, clothes | | Diarrhœa | 2 | Liver, abscess of | 1 |
| taking fire | 2 | " chronic | 2 | " congestion of | 1 |
| " brain concussion | 2 | Diphtheria | 7 | " enlargement of | 1 |
| " chloroform surgi- | | Dropsy general | 1 | " and stomach, con- | |
| cal operation | 1 | " of abdomen | 1 | gestion of | 1 |
| " crushed by bridge | 1 | " of chest | 2 | Lungs, congestion of | 5 |
| " city railroad cars | 1 | Drowned | 2 | " hemorrhage of | 4 |
| " drowned | 3 | Dysentery | 3 | Manslaughter | 1 |
| " fall | 4 | Embolia from cardiac | | Mortification of leg | 1 |
| " fracture of skull | 1 | inflammation | 1 | Measles | 14 |
| " poison | 1 | Enteritis | 7 | " and complications | 10 |
| " run over by team | 2 | Erysipelas | 1 | Metroperitonitis | 1 |
| " steam railroad cars | 3 | Fever congestive | 1 | Meningitis | 5 |
| " suffocation | 2 | " puerperal, | 3 | " cerebro-spinal | 2 |
| Apoplexy | 5 | " remittent | 1 | " tubercular | 2 |
| Asthma | 1 | " scarlet | 3 | Old age | 9 |
| Atelectasis pulmonium | 1 | " " & complicat's | 3 | Ostitis of tibia | 1 |
| Bowels, anasarca of | 1 | " " malignant | 1 | Œdema pulmonum | 1 |
| " congestion of | 1 | " typhoid | 9 | Pericarditis | 1 |
| Brain, compression of | 1 | Gastromalaxia | 1 | Peritonitis | 2 |
| " congestion of | 7 | Gastritis | 4 | Pneumonia | 42 |
| " concussion and | | Gastro-enteritis | 2 | " broncho | 2 |
| meningitis | 1 | Hæmatemesis | 1 | " and complications | 2 |
| " inflammation of | 7 | Hæmorrgica purpura | 1 | " typhoid | 3 |
| Bronchitis | 4 | Heart disease | 3 | Scrofula | 3 |
| " capillary | 5 | " paralysis of | 2 | Small-pox | 1 |
| Cachexia malaria | 2 | " hypertrophy of | 1 | Spinal cord, disease of | 1 |
| Cancer of breast | 1 | " organic disease of | 1 | Suicide by shooting | 2 |
| " of shoulder | 1 | " valvular disease of | 7 | " by drowning | 1 |
| " of stomach | 1 | Hepatitis | 1 | Syphilis hereditary | 1 |
| " of uterus | 3 | Hydrocephalus | 2 | Tabes mesenterica | 15 |
| Cerebral spinal irritation | 1 | " acute | 5 | Teething | 4 |
| Childbirth | 1 | Injury of vertebra and | | " and complications | 1 |
| Cholera infantum | 1 | paralysis | 1 | Whooping-cough | 7 |
| Consumption | 48 | Insanity | 1 | | |
| Convulsions | 39 | Inanition | 12 | Total | 421 |
| Croup | 9 | Intestines, gangrene of | 1 | | |
| " membraneous | 2 | Kidneys, Bright's dis- | | Premature births | 15 |
| Cynanche trachealius | 1 | ease of | 1 | Still births | 61 |
| Cystitis chronic | 1 | Laryngismus stridulas | 1 | | |
| Debility, general | 3 | Laryngitis | 4 | Total | 76 |

### AGES.

| | | | | | |
|---|---|---|---|---|---|
| Under 1 | 130 | 10 to 20 | 15 | 70 to 80 | 11 |
| 1 to 2 | 44 | 20 to 30 | 49 | 80 to 90 | 2 |
| 2 to 3 | 18 | 30 to 40 | 39 | 90 to 100 | 0 |
| 3 to 4 | 17 | 40 to 50 | 25 | 100 to 102 | 1 |
| 4 to 5 | 6 | 50 to 60 | 27 | | |
| 5 to 10 | 23 | 60 to 70 | 14 | Total, | 421 |

| | | | | | |
|---|---|---|---|---|---|
| Males, | 226 | Females, | 195 | Total, | 421 |
| Single, | 298 | Married | 123 | Total, | 421 |
| White, | 409 | Colored, | 12 | Total, | 421 |

COMPARISON.

Deaths in April, 1871,--421 | Deaths in April, 1870,-- 495 | Decrease,---- 74
Deaths in Mar., 1871,------------ 472 | Decrease, ------------------- 51

NATIVITY.

| | | | | | |
|---|---|---|---|---|---|
| Austria | 0 | England | 6 | New Brunswick | 0 |
| Bohemia | 4 | France | 2 | Norway | 5 |
| Canada | 4 | Germany | 52 | Poland | 3 |
| Chicago, Native | 77 | Holland | 1 | Switzerland | 1 |
| Chicago, Foreign | 135 | Ireland | 45 | Scotland, | 3 |
| U. S., other parts | 68 | Italy | 1 | Sweden | 9 |
| Denmark | 3 | New Foundland | 0 | Unknown | 2 |
| | | | | Total, | 421 |

MORTALITY BY WARDS FOR THE MONTH.

| Wards. | Mortality. | Pop. in 1870. | One death in |
|---|---|---|---|
| 1 | 6 | 6,531 | 1088 |
| 2 | 13 | 14,338 | 1103 |
| 3 | 20 | 16,805 | 840 |
| 4 | 15 | 12,178 | 812 |
| 5 | 12 | 11,605 | 967 |
| 6 | 15 | 19,486 | 1299 |
| 7 | 11 | 13,849 | 1259 |
| 8 | 26 | 22,994 | 887 |
| 9 | 39 | 27,278 | 699 |
| 10 | 24 | 13,750 | 573 |
| 11 | 23 | 14,988 | 652 |
| 12 | 8 | 13,976 | 1747 |
| 13 | 5 | 8,943 | 1788 |
| 14 | 9 | 9,076 | 1008 |
| 15 | 43 | 20,382 | 474 |
| 16 | 16 | 13,975 | 873 |
| 17 | 30 | 17,118 | 571 |
| 18 | 22 | 17,069 | 776 |
| 19 | 6 | 8,738 | 1456 |
| 20 | 7 | 13,628 | 1947 |

| Mortality. | |
|---|---|
| Accidents | 23 |
| Burlington Slip | 1 |
| County Hospital | 13 |
| Foundling Home | 10 |
| Home for Friendless | 3 |
| Immigrants | 2 |
| Illinois Central Slip | 1 |
| Marine Hospital | 1 |
| Mercy Hospital | 2 |
| Hospital Alexian Brothers | 4 |
| Manslaughter | 1 |
| St. Joseph Orphan Asylum | 7 |
| Suicide | 3 |
| Total | 421 |

Mean Thermometer for month, 52⅜°; Rain, 2.950 inches; Deaths daily, 14.

